# Image Significance in the Diagnosis of Another Case of Herpetic Encephalitis

**DOI:** 10.7759/cureus.53883

**Published:** 2024-02-08

**Authors:** José Pedro Manata, Bernardo Silva, Mafalda Sousa, João Matos Costa

**Affiliations:** 1 Internal Medicine, Hospital Distrital de Santarém, Santarém, PRT

**Keywords:** acyclovir, temporal lobes, acute necrotizing encephalitis, hsv-1, encephalitis

## Abstract

Herpes simplex virus 1 (HSV-1) causes necrotizing encephalitis, usually located in the temporal lobes and with a high mortality rate if not diagnosed and treated early. Cranial computed tomography (CT) scan, although not very sensitive, can help by highlighting hemorrhagic foci and edema in the frontotemporal lobes, given the tropism of the virus for these areas. We present the case of a 70-year-old male who came to the emergency department (ED) with fever and confusion. Despite an unclear cerebrospinal fluid (CSF) result, the CT scan showed a spot of hypodensity in the mesial aspect of the left temporal lobe. He was given 21 days of intravenous acyclovir, and his neurological condition normalized. These cranial CT alterations, although not pathognomonic, indicate a strong suspicion of herpetic encephalitis.

## Introduction

Encephalitis is an inflammation of the brain parenchyma, and the etiology is often unknown. The cases with a known cause are usually of viral etiology. The most common etiological agents are herpesviruses (herpes simplex virus 1 {HSV-1} and herpes simplex virus 2 {HSV-2}), with varicella-zoster virus (VZV), enteroviruses, and arboviruses accounting for the remainder [1-4]. Around 90% of the cases of herpesvirus encephalitis in adults are due to HSV-1, with HSV-2 being associated with neonatal encephalitis [5-7].

Herpes encephalitis (HE) is a cause of acute necrotizing encephalitis, which is usually asymmetrically located in the orbitofrontal and temporal lobes, and has a high mortality rate of 70% if not treated early [7-9].

HSV-1 is the only viral agent with tropism for the temporofrontal lobes, capable of causing necrotic and hemorrhagic lesions. Cranial computed tomography (CT) scan is not very sensitive, but it can reveal the presence of localized edema, hypodense lesions, and hyperdense lesions such as hemorrhages [2,5].

The diagnosis is consolidated using magnetic resonance imaging (MRI) and polymerase chain reaction (PCR) detection of herpesviruses in the cerebrospinal fluid (CSF). The electroencephalogram (EEG), when available, is a tool to take into account since up to 80% of the cases show alterations [1,8].

## Case presentation

We report the case of a 70-year-old male who was referred to the emergency department (ED) by his doctor for headaches, confusion, and fever that had been going on for four days. When he was seen in the ED, he was confused, with vague and hesitant speech. The neurological examination was normal, with no meningeal signs.

**Figure 1:**
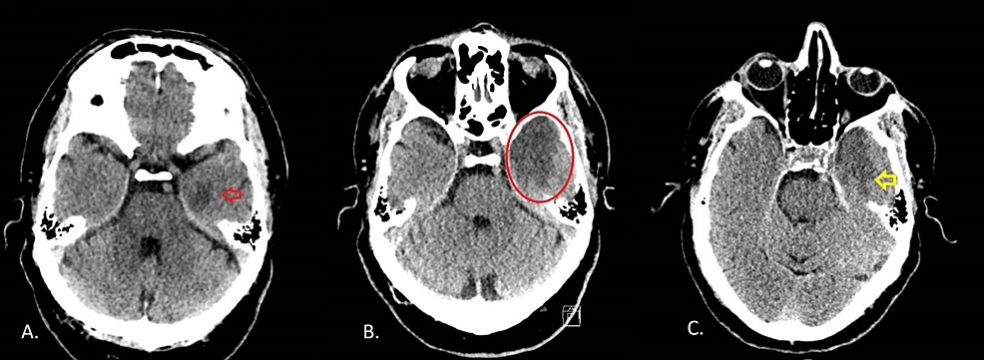
Time course of herpetic encephalitis (A) Hypodensity in the left mesial temporal area (red arrow). (B) Eighth day of hospitalization with worsening of the hypodensity focus (red circle). (C) Improvement in the area involved, already on the treatment with intravenous acyclovir (yellow arrow)

In the emergency department, he underwent a brain CT scan and lumbar puncture. As can be seen in Figure 1A, the brain CT scan showed, in addition to a mild pattern of microangiopathic chronic ischemic leukoencephalopathy, the presence of hypodensity in the left mesial temporal area, reflecting edema and correlating with possible encephalitis. The lumbar puncture (LP) revealed a slightly cloudy and pinkish CSF, with 50.3 mg/dL of protein and 79 mg/dL of glucose. The total cell count revealed 81 neutrophils and many erythrocytes (Table 1).

**Table 1:** Results of two lumbar punctures (LP) MN, mononuclear cells; PMN, polymorphonuclear cells; ED, emergency department

Cerebrospinal fluid					
	Color	Differential cells	Other cells	Proteins (mg/dL)	Glucose (mg/dL)
First LP: admission to the ED	Cloudy and pink	81 PMN	RBCs	50.3	79
Second LP: eighth day of hospitalization	Clear	155 MN + 2 PMN	-	49.5	70

Given the results mentioned above and the confusional state, he is hospitalized on the assumption of bacterial meningoencephalitis; therefore, vancomycin, ceftriaxone, and ampicillin were started.

During hospitalization and given the negative cultures, ampicillin and vancomycin were suspended, while ceftriaxone was maintained.

On the eighth day of hospitalization, he had a new febrile peak of 38.2°C, a new cranial CT scan was repeated, and a body CT scan was also performed to rule out an abscess. New blood cultures and urocultures were carried out.

The reassessment CT scan showed a worsening of the image, with a greater extension of the hypodense area in the left temporal region (Figure 1B).

The clinical case was discussed with the neurology department, which decided that, given the location of the lesion and the febrile condition, a new LP should be performed, acyclovir should be started, and antibiotic therapy with ceftriaxone should be maintained. They also suggested a brain MRI for differential diagnosis.

The new LP showed clear, colorless CSF; protein of 49.5 mg/dL; glucose of 70.0 mg/dL; and a total cell count of 157, with 155 mononuclear cells and two polymorphonuclear cells (Table 1). The CSF test for herpes simplex virus 1 (HSV-1) was positive. MRI confirmed the presence of extensive cortico-subcortical signal alterations in the left temporal and insular regions, with hypersignal in T2 and the obliteration of the adjacent sulci, suggestive aspects of herpetic encephalitis.

He completed 21 days of acyclovir (Figure 1C), with good clinical and imaging response.

## Discussion

The presumption of the initial diagnosis in the ED was based essentially on the clinical picture and the assessment of a CSF with a predominance of polymorphonuclear cells, disregarding the alterations of the brain CT scan, which, although not pathognomonic, are of strong suspicion for the diagnosis.

Herpes simplex infection is common, but encephalitis caused by this virus is rare. The worldwide incidence is around 2-4 cases/1,000,000, and there is a bimodal distribution with a peak incidence up to the age of three and then again in adults over the age of 50, with both sexes being equally affected.

The mechanisms by which HSV-1 gains access to the central nervous system are still not clear. The most likely route includes retrograde transport through the olfactory nerves, in which the pathways do not route through the thalamus but connect directly to the frontal and mesiotemporal lobes.

Its tropism for the temporal lobes can cause necrotic and hemorrhagic lesions, leading to fulminant necrotizing encephalitis, which has a high mortality rate if not treated early. These lesions are usually asymmetrical and located in the orbitofrontal and temporal lobes with the involvement of the cingulate and insular cortex [5,7].

Empirical treatment should be started as soon as viral encephalitis is suspected. The administration of acyclovir up to the fourth day of illness considerably reduces mortality. The use of acyclovir intravenously at a dose of 10-15 mg/kg/day every eight hours for a period of 14-21 days has become the treatment of choice. The low availability of oral acyclovir contraindicates its use in these situations [1,3,6].

## Conclusions

The imaging review during hospitalization was decisive in changing the therapeutic attitude. It should be emphasized that the neuroimaging examinations served to guide the diagnosis, enabling early intervention with the institution of antiviral therapy, which was crucial in terms of possible sequelae or even the fatal evolution of this case.
